# Daphnoretin inhibited SCI-induced inflammation and activation of NF-κB pathway in spinal dorsal horn

**DOI:** 10.18632/aging.205893

**Published:** 2024-06-05

**Authors:** Jiazhang Wu, Fengfei Lin, Bin Chen

**Affiliations:** 1 Department of Orthopaedics, Fuzhou Second General Hospital, School of Clinical Medicine, Fujian Medical University, Fuzhou 350007, China; 2 Department of Orthopaedics, Fuzhou Second Hospital of Xiamen University, School of Medicine, Xiamen University, Fuzhou 350007, China; 3 Fujian Provincial Clinical Medical Research Center for First Aid and Rehabilitation in Orthopaedic Trauma, Fuzhou Trauma Medical Center, Fuzhou 350007, China

**Keywords:** spinal cord injury, daphnoretin, NF-κB, microglia, neuroinflammation

## Abstract

Objective: Spinal cord injury (SCI) is a devastating disease for which there is no safe and effective treatment at present. Daphnoretin is a natural discoumarin compound isolated from *Wikstroemia indica* with various pharmacological activities. Our study aimed to investigate the role of Daphnoretin in NF-κB pathway activation and inflammatory response after SCI.

Methods: A mouse SCI model was constructed, and the Basso Mouse Scale Score and subscore were used to evaluate the effect of Daphnoretin on the movement capacity of mice. The effect of Daphnoretin on the activation of glial cells in the mouse model and BV2 cells was observed by immunofluorescence. PCR and ELISA were used to detect the expression of inflammatory factors, and Western blot was performed to detect the protein expression associated with NF-κB pathway.

Results: Daphnoretin inhibited the loss of movement ability and the activation of glial cells in mice after SCI, and it also inhibited the activation of NF-κB pathway and the expression of inflammatory factors TNF-α and IL-1β *in vivo* and *in vitro*.

Conclusions: Daphnoretin can inhibit the activation of NF-κB pathway and the inflammatory response induced by SCI. Our study demonstrates the potential of Daphnoretin on clinical application for the treatment of SCI.

## INTRODUCTION

Spinal cord injury (SCI) is a devastating disease, and there were approximately 2-3 million people suffering from SCI-related disability worldwide. SCI is often caused by accidents, falls and trauma [1, 2]. It can lead to motor, sensory and autonomic neurological dysfunction and has complex pathophysiological mechanisms, so it remains difficult to cure [3]. Neuropathic pain (NP) is a common complication in SCI patients, with a prevalence of approximately 58% [2, 4, 5]. SCI results in sustained activation of microglia and astrocytes in the spinal cord, sensitizing spinal dorsal horn neurons by releasing neurotransmitters, pro-inflammatory cytokines, and chemokines [6]. These inflammatory factors can exacerbate hyperalgesia and increase blood brain barrier (BBB) permeability, leading to programmed cell death [7]. Glial cell-mediated inflammatory response plays a key role in the occurrence and development of NP [8]. In particular, activated microglia can cause nerve cell death by secreting interleukin (IL), tumor necrosis factor (TNF) and interferon (INF), and play an important role in initiating and maintaining NP and remodeling the nervous system structure [8, 9].

Daphnoretin is a natural discoumarin compound isolated from *Wikstroemia indica* [10]. It has multiple pharmacological effects of antitumor. Previous studies have found that Daphnoretin could inhibit the proliferation, migration and viability of colon cancer cells [11], malignant melanoma cells [12], lung cancer cells [13] and cervical cancer cells [14] *in vitro*. And it can regulate the differentiation and maturation of DCs by mediating p-JUk activity [15]. Meanwhile, Daphnoretin was found to have anti-anxiety [16], anti-epilepsy [17] and antiviral [18] activities. Recently, studies revealed that Daphnoretin showed dose-dependent protective effects in OA by inactivating ERS and NLRP3 inflammasome [19]. But whether it can affect nerve inflammation is still unclear.

It has been reported that nuclear factor-κB (NF-κB) is a ubiquitous transcription factor, and its change is a key mechanism of SCI-induced secondary injury [20–22]. Not only is it a transcriptional activator of inflammatory mediators, it can also regulate neurotransmission [23, 24]. Activated microglia can regulate NF-κB expression [25, 26]. At the same time, NF-κB can also mediates the inflammatory response of microglia cells [27, 28]. Numerous studies have highlighted the crucial role of NF-κB in the pathogenesis of SCI-induced pain [29–31]. Meanwhile, Daphnoretin has shown promise in attenuating inflammation in various diseases [19]. Given the central role of inflammation in SCI-induced pain, it is plausible to hypothesize that Daphnoretin may mitigate pain associated with SCI by modulating NF-κB-mediated inflammatory response. Therefore, this study aims to investigate the effect of Daphnoretin in regulating glia cells and NF-κB pathway activation after SCI, providing valuable insights into novel treatment strategies for this challenging condition (Graphic abstract).

**Graph abstract:**
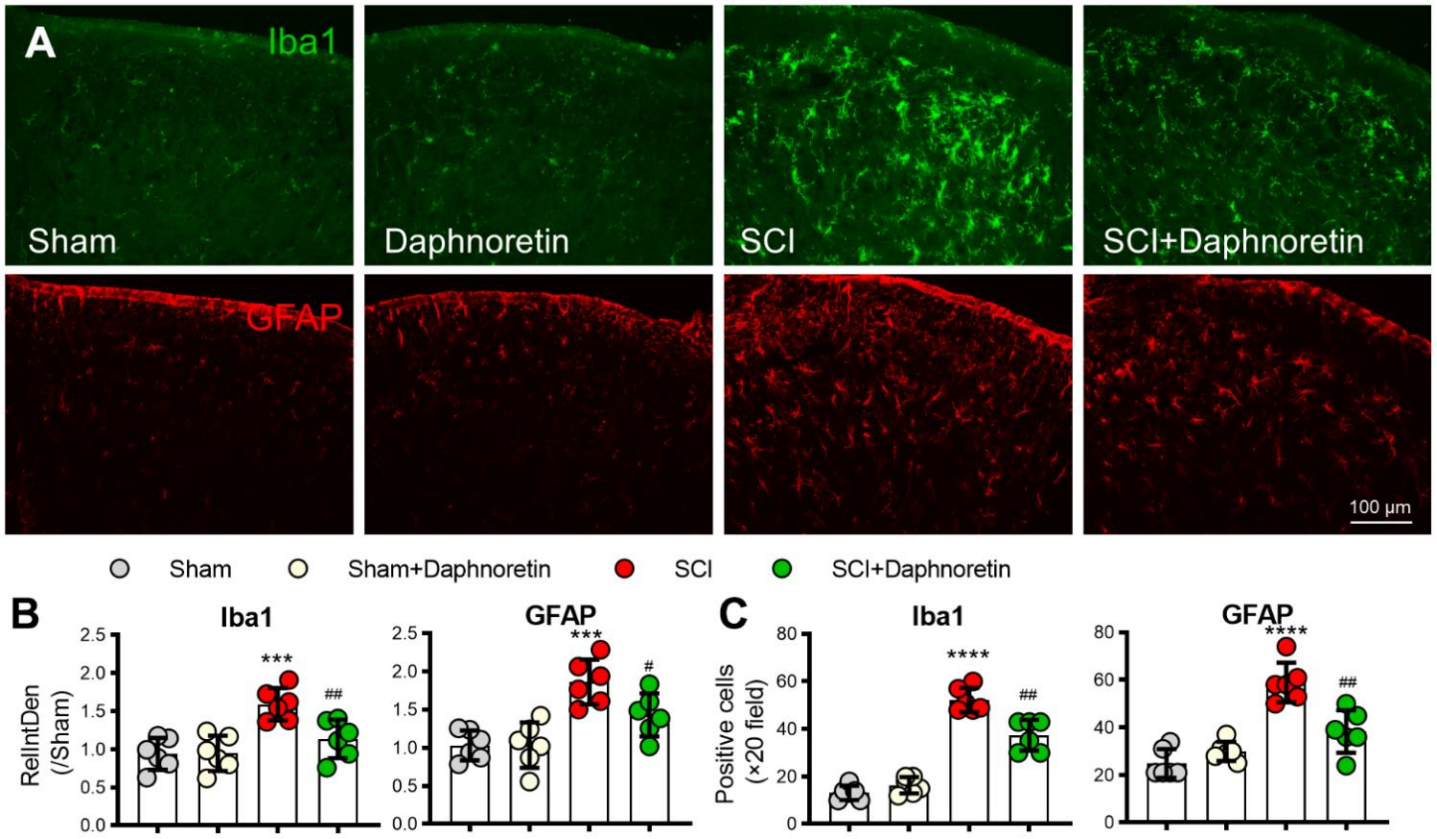
Overview of Daphnoretin’s mechanism of action in SCI.

## RESULTS

### Daphnoretin attenuated motor function of mice after SCI

**Figure 1:**
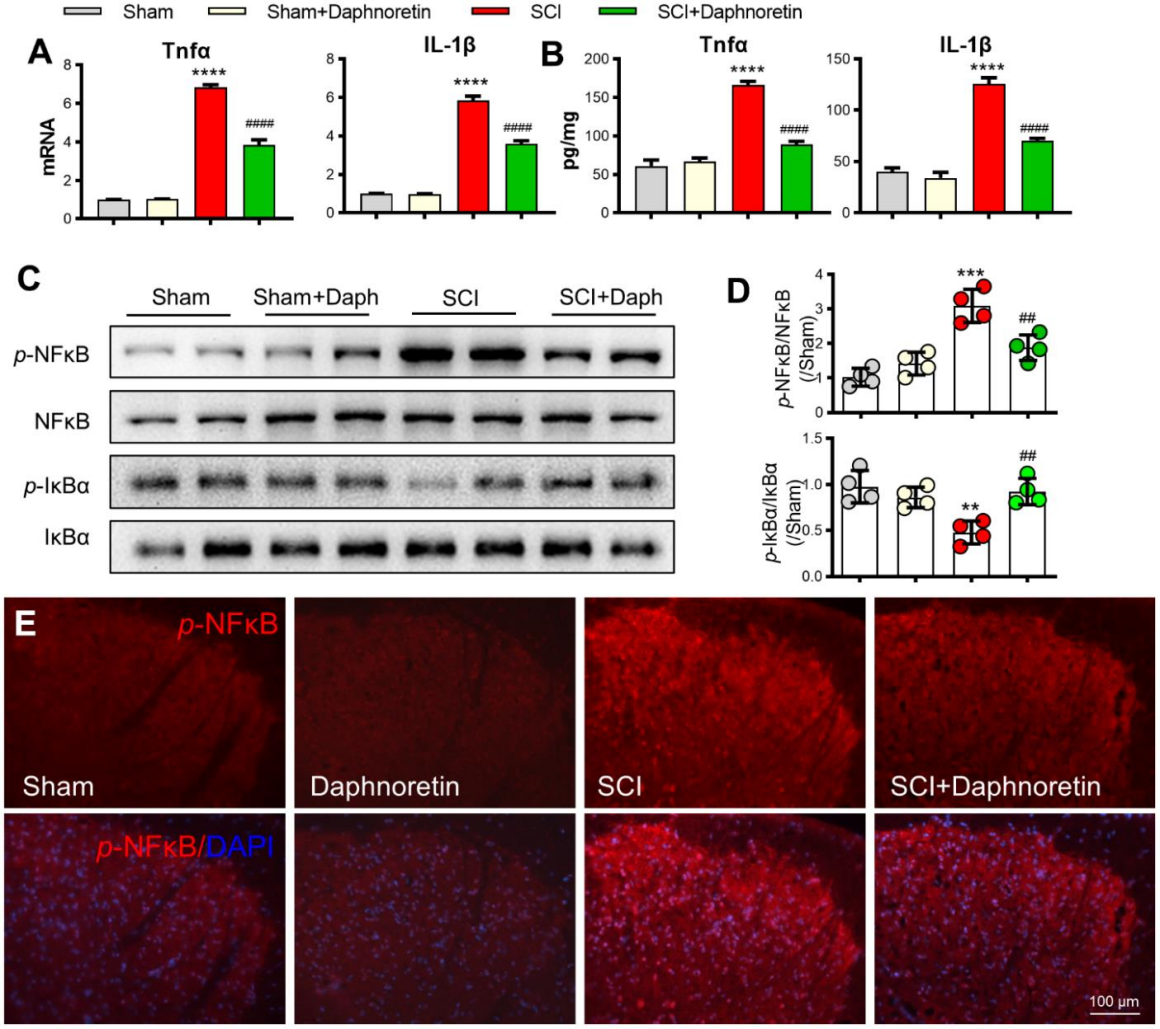
**Daphnoretin attenuated motor function of mice after SCI.** (**A**) Experimental scheme of Daphnoretin treatment after SCI. (**B**) Effects of Daphnoretin on BMS score and BMS subscore of mice after sham or SCI. n=6 mice/group, ****P < 0.0001 vs Sham group, ###*P* < 0.001, ####*P* < 0.0001 vs SCI group. The data were analyzed by two-way ANOVA with Tukey’s test.

To evaluate the effect of Daphnoretin on motor function after SCI. Daphnoretin (200 mg/kg, 200 μl) was intraperitoneally injected immediately after SCI, once a day for a total of 3 times. BMS Score and BMS subscore were assessed at -1, 1, 3, 7, 14, 21, 28, 40, and 60 days after SCI (Figure 1A). Intraperitoneal injection of Daphnoretin had no effect on the motor function of the Sham group, and the BMS score and BMS subscore remained full until the 60^th^ day. Motor function was severely impaired immediately after SCI (*P* < 0.0001), while intraperitoneal injection of Daphnoretin significantly improved BMS score and BMS subscore from day 7 (Figure 1B).

### Daphnoretin inhibited SCI-induced glial cell activation in spinal dorsal horn

**Figure 2:**
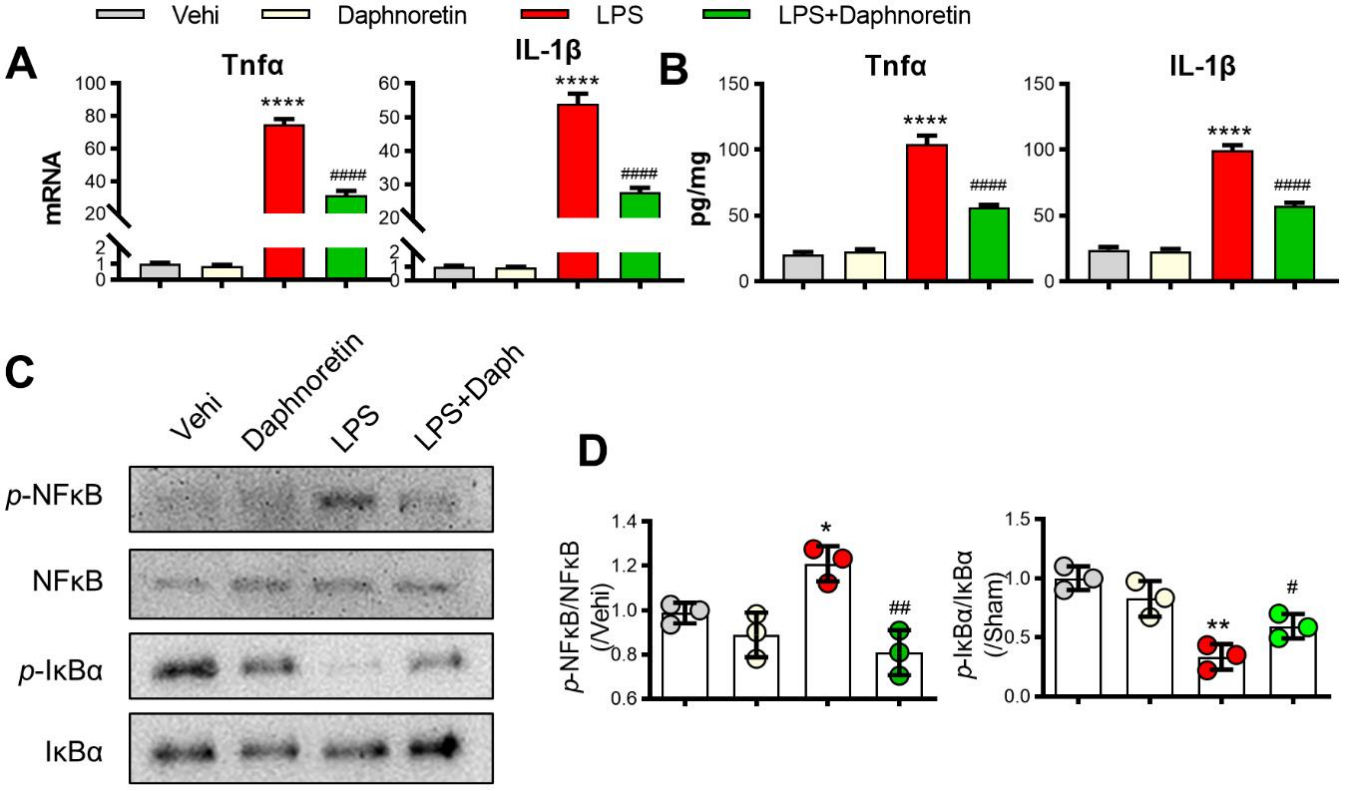
**Daphnoretin can inhibit SCI-induced glial cell activation in spinal dorsal horn.** (**A**) Immunofluorescence showed the effect of Daphnoretin on glial cells in spinal dorsal horn after SCI. DAPI (blue), Iba1 (microglia green), GFAP (astrocytes, red). Scale bars: 100 μm. (**B**, **C**) The changes of fluorescence intensity (**B**) and number of positive cells (**C**) in each group were statistically analyzed. n = 3 mice/group, 2 sections/mice, ****P < 0.0001 vs Sham group, ###P < 0.001, ####P < 0.0001 vs SCI group, the data were analyzed by one-way ANOVA with Tukey’s test.

Since the activation of glial cells plays a key role in the occurrence and development of pain, immunofluorescence was performed on the lumbar spinal cord of mice 7 days after SCI to observe the effect of Daphnoretin on glial cells in spinal dorsal horn. SCI resulted in significant activation of microglia (Iba1 labeled, green) and astrocytes (GFAP labeled, red) (*P* < 0.0001). Daphnoretin had no effect on the activation of glial cells in the Sham group, but significantly inhibited the activation of glial cells after SCI (*P* < 0.0001) (Figure 2).

### Daphnoretin inhibited SCI-induced inflammation and activation of the NF-κB pathway in spinal dorsal horn

**Figure 3:**
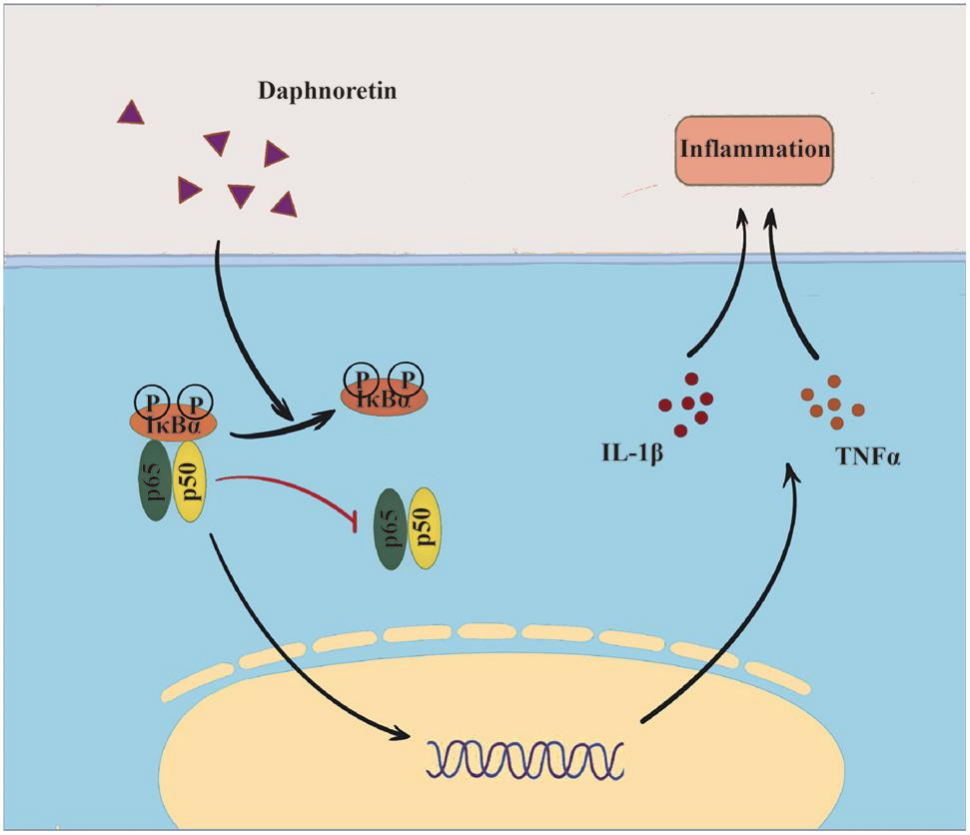
**Daphnoretin can inhibit SCI-induced inflammation and activation of the NF-κB pathway in spinal dorsal horn.** (**A**) The mRNA expression of TNF-α and IL-1β in spinal dorsal horn was measured by PCR. (**B**) The protein expression of TNF-α and IL-1β in spinal dorsal horn was measured by ELISA. (**C**, **D**) The protein expression of *p*-NF-κB, NF-κB *p*-IκBα and IκBα was examined by Western blot. n = 4 /group. (**E**) Immunofluorescence showed the effect of Daphnoretin in spinal dorsal horn after SCI. DAPI (blue), *p*-NF-κB (red). **P < 0.01, ***P < 0.001****P < 0.0001 vs Sham group, ##P < 0.01, ####P < 0.0001 vs SCI group, the data were analyzed by one-way ANOVA with Tukey’s test.

After analyzing the PCR and ELISA results, we found that TNF-α and IL-1β expressions in spinal dorsal horn were significantly increased after 7 days of SCI, and intraperitoneal injection of Daphnoretin inhibited the inflammatory response in spinal dorsal horn (Figure 3A, 3B). Meanwhile, Western blot analysis showed that NF-κB Pathway was activated after SCI, and the expression of *p*-NF-κB/ NF-κB was significantly increased, while *p*-IκBα/ IκBα expression was decreased. The increase of *p*-NF-κB and decrease of *p*-IκBα were inhibited by intraperitoneal injection of Daphnoretin (Figure 3C, 3D). Immunofluorescence results also showed that Daphnoretin could inhibit the increase of *p*-NF-κB after SCI (Figure 3E).

### Daphnoretin inhibited LPS-induced NF-κB pathway activation and inflammation in BV2 cells

**Figure 4:**
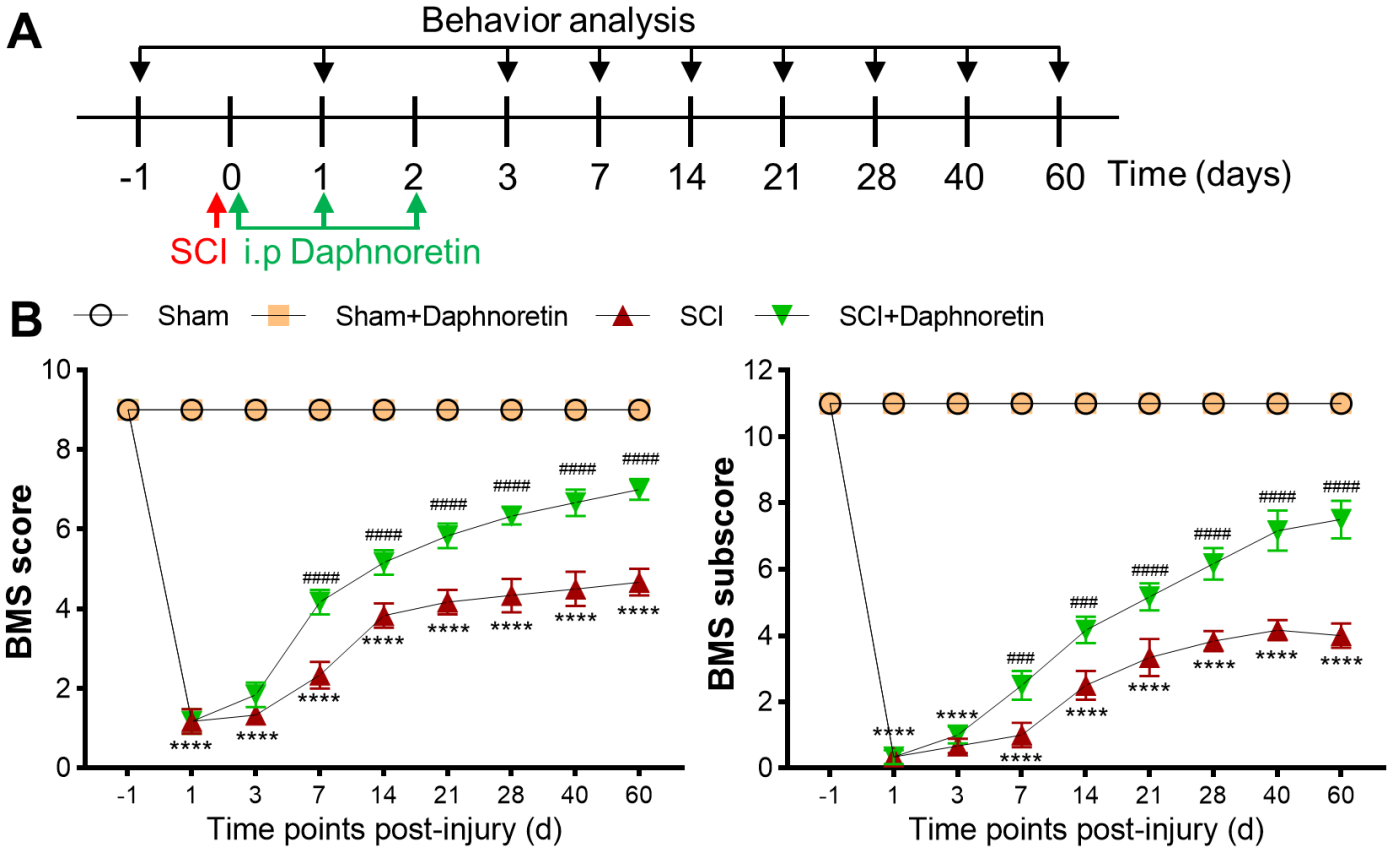
**Daphnoretin inhibited LPS-induced NF-κB pathway activation and inflammation in BV2 cells.** (**A**) The mRNA expression of TNF-α and IL-1β in BV2 cells were measured by PCR. (**B**) The protein expression of TNF-α and IL-1β in BV2 cells were measured by ELISA. (**C**, **D**) The protein expression of *p*-NF-κB, NF-κB *p*-IκBα and IκBα were examined by Western blot. n = 3 /group, *P < 0.05, ***P < 0.01, ****P < 0.0001 vs Sham group, #P < 0.05, ##P < 0.01, ####P < 0.0001 vs SCI group, the data were analyzed by one-way ANOVA with Tukey’s test.

To further verify the effect of Daphnoretin on glial cell inflammation and NF-κB pathway, we tested the effect of Daphnoretin on BV2 cells *in vitro*. First, PCR and ELISA results showed that Daphnoretin inhibited LPS-induced significant increase in the expression of TNF-α and IL-1βin BV2 cells *in vitro* (Figure 4A, 4B). The inhibitory effect of Daphnoretin on NF-κB pathway was further demonstrated by WB, in which Daphnoretin inhibited the increase of *p*-NF-κB/NF-κB and the decrease of *p*-IKBα/IKBα (Figure 4C, 4D).

## DISCUSSION

Spinal cord injury (SCI) can be divided into primary injury and secondary injury. Since secondary injury is reversible, how to inhibit the loss of motor function and inflammation caused by SCI is the main direction of current research [32]. Amyelotrophy and neurodegeneration following SCI could initiate neurodegeneration [33]. Microglia are rapidly activated after SCI and release a large number of inflammatory factors, such as TNF-α and IL-1β [34]. TNF can aggravate the inflammatory response after injury by increasing the activity of nitric oxide synthase and inducing chemotaxis of inflammatory cells [35]. IL-1β is also released after SCI and exacerbates inflammatory response [36]. Astrocytes can also have adverse effects through the process of reactive astrocyte proliferation [37]. The effect of Daphnoretin on neuroinflammation has not been studied yet. However, in current study we found that Daphnoretin not only could attenuate the motor function of mice after SCI, but also inhibited the activation of microglia and astrocytes in spinal dorsal horn and the expression of inflammatory factors TNF-α and IL-1β.

NF-κB pathway is involved in cell survival, apoptosis, proliferation and differentiation, and has been considered as the main pathophysiological mechanism of secondary SCI inflammatory response and an important target for therapeutic drugs to alleviate SCI inflammation [38, 39]. NF-κB pathway regulates multiple aspects of innate and adaptive immune function and acts as a key mediator of inflammatory response [40]. In non-inflammatory conditions, inactive NF-κB is sequestrated in the cytoplasm, and p-NF-κB can be released by IκB and translocated into the nucleus when certain conditions activate IKK (IκB kinase) [26].

NF-κB can directly induce the production of pro-inflammatory cytokines such as TNF-α and IL-6, and it can also induce the secretion of IL-β’s inactive precursor pro-IL-1β, which will be subsequently cleaved by active caspase-1 for maturation. At the same time, these pro-inflammatory factors also activate NF-κB. Under this circumstance, NF-κB pathway together with above mentioned pro-inflammatory factors upregulate each other and increase inflammatory responses, and therefore take important part in the occurrence and development of SCI [41, 42]. It also affects nerve regeneration and synapses formation by regulating the level of anti-apoptotic proteins [21, 43]. Therefore, the inhibition of the synergistic effect of NF-κB and inflammatory factors is an important direction to investigate novel medicines for the treatment of inflammatory diseases. Daphnoretin has been proven capable of regulating Akt11 signaling pathway [11] and PKC expression level [44], and could also activate caspase-3 and caspase-9 to promote HeLa cell apoptosis in a mitochondria-mediated manner [14]. However, whether these functions of Daphnoretin is related to NF-κB pathway has not been studied yet. Our studies found the inhibition effect of Daphnoretin on inflammation and NF-κB activation not only in the spinal dorsal horn, but also in microglia cell line BV2 cells *in vitro*.

However, this study also has some limitations. We identified the involvement of PI3K-Akt pathway in IVDD, but by how it was deactivated and whether it has correlations with the GPX7-regulated cellular aging remain unclear. GNF-5837 is currently being regarded as a potent pan-TRK inhibitor. And previous studies have reported that TRK families could inhibit cell apoptosis and promote the cell cycle progression by activating PI3K-Akt pathway, which suggests that GNF-5837 may have potential function in inducing G0/G1-phase arrest and apoptosis. Although our results confirmed that GNF-5837 could alleviating IVDD by increasing GPX7 expression, its side effects still need to be further examined. As such, before its administration to humans to treat aging-related disease, several issues and hurdles need to be addressed, including determining the optimal dose and treatment duration.

While this study provides valuable insights into the potential therapeutic effects of Daphnoretin in SCI, it’s important to acknowledge several limitations. Firstly, the optimal dosage and potential side effects of Daphnoretin have yet to be fully elucidated. Although our findings demonstrate promising outcomes in preclinical models, translating these results to clinical settings requires careful consideration of dosage regimens and safety profiles. Moreover, the complexity of SCI pathology and individual patient variability present significant challenges in determining the precise therapeutic dose of Daphnoretin. Further investigations, including dose-ranging studies and comprehensive safety assessments, are warranted to address these concerns and facilitate the development of Daphnoretin-based therapies for SCI patients. Therefore, while our findings lay the foundation for the potential clinical application of Daphnoretin in SCI treatment, additional research is required before it can be considered a viable treatment option for SCI patients.

In conclusion, Daphnoretin can inhibit the activation of NF-κB pathway to inhibit activated glial cells and reduce the release of inflammatory factors *in vivo* and *in vitro*. And has potential on clinical application for the treatment of SCI.

## MATERIALS AND METHODS

### Animals and ethics statement

Male C57BL/6 mice (8-10 week, weighing 22±2 g) were purchased from Fuzhou Second General Hospital (Fuzhou, China) and housed in a specific-pathogen-free (SPF) animal facility with a 12-h light/dark cycle at a controlled temperature (22 ± 2° C), humidity of 40%–60%, and ad libitum access to water and food. Same sex mice were housed together in individually ventilated cages with four mice per cage. The Committee for Animal Care and Use at Graduate Fuzhou Second General Hospital duly passed all experiments performed on animals (2019701172).

### Materials and drugs

Daphnoretin (PHL83490) and Lipopolysaccharide (LPS, L4516) were from Sigma-Aldrich (St. Louis, MO, USA). Antibody for Iba1 (ab5076) was from Abcam (Cambridge, MA, USA). Antibodies for *p*-NF-κB p65 (#3033), NF-κB p65 (#4764), *p*-AMPK (#2535), AMPKα (#2532), GFAP (#3670) were from Cell Signaling Technology (Beverly, MA, USA).

### Spinal cord injury model

All animal experiments took place at Fuzhou Second General Hospital. Male C57BL/6 mice (8-10 week, weighing 22±2 g) strictly follow animal ethics. All mice were randomly grouped and intraperitoneally anesthetized with 1% pentobarbital sodium (50mg/kg, P3761, Sigma-Aldrich, St. Louis MO, USA). SCI model was established on mice according to the methods reported previously [45]. The mice were randomly assigned to different groups with 6 mice in each group. The mice were anesthetized with oxygen containing 1.5% isoflurane and fixed on the operating table in a prone position.

Disinfection and an 30mm-length incision were made in a radius around the back of T12. The subcutaneous tissue was incised, and the muscular tissue was carefully blunt dissected along the vertebral body layer by layer until to the lamina. The spinal cord was exposed by laminectomy at the level of T12. The right spinal cord was transected by microsurgical scissors. And then a 11-gauge blade was used to cut through the spinal cord incision to ensure complete transection of the right spinal cord. Finally, the skin was sutured layer by layer and disinfected. The above-mentioned operations were all performed under a stereo microscope. In the sham operation group, only laminectomy was performed without damaging the spinal cord. Finally, sodium pentobarbital injection was used for euthanasia: 150-200mg/kg of the drug was injected intraperitoneally.

### Behavioral test

On day -1,1,3,7,14,21,28,40, and 60 after spinal cord injury, mice in each group were placed on an open plane, and the recovery of the right hind limb motor function of mice after spinal cord injury was evaluated by Basso Mouse Scale (BMS) score and the BMS subscore [46]. No ankle movement and complete paralysis of hind limbs were defined as 0 point; 1 point for slight ankle movement; mild trunk instability scored 8 points and no movement disorder scored 9 points. The means of the BMS primary score and the BMS subscore were recorded, and the mean values were calculated as the final score value. Two blinded investigators who have been well trained and experienced in BMS assessment assessed randomly selected mice in an open area within 4 minutes.

### Cell culture

The mouse microglia BV2 cell line was purchased from the Procell Life Science and Technology Co., Ltd. (CL-0493A; Wuhan, China) and cultured in Dulbecco’s modified Eagle medium (DMEM, Gibco, Waltham, MA, USA) supplemented with 10% fetal bovine serum (FBS, Gibco, Brazil) and 1% penicillin-streptomycin (PS) at 37° C in a 5% CO_2_ incubator. The cells were passaged or seeded in plates when grown to about 80% confluency. After 24 hours of plating, the cells were incubated with serum-free DMEM for 4 hours, and then stimulated with 100ng/mL LPS for 1 or 24 hours after 30 μM Daphnoretin pretreatment for 30 minutes.

### Real-time quantitative PCR (RT-qPCR)

The cells were lysed by Trizol, and total RNA was extracted and reverse transcribed into cDNA. Finally, the mRNA expression of IL-1β and TNF-α was analyzed by fluorescence quantitative PCR reagent with GAPDH as internal reference. The cycle threshold (CT) value in this study was 20. The primers were synthesized by Shanghai Sangon Biological Company (Shanghai, China). All the primer sequences used in this study are listed in Table 1.

**Table 1:** Primer sequences for real-time quantitative PCR.

**Gene**	**Forward primer sequences**	**Reverse primer sequences**
**TNF-α**	5-CCTGTAGCCCACGTCGTAG-3	5-GGGAGTAGACAAGGTACAACCC-3
**IL-1β**	5-CTGTGACTCATGGGATGATGATG-3	5-CGGAGCCTGTAGTGCAGTTG-3
**GAPDH**	5-GACAGTCAGCCGCATCTTCTT-3	5′-AATCCGTTGACTCCGACCTTC-3

### ELISA

IL-1β and TNF-α levels in mouse spinal cord and BV2 cell culture supernatants were quantified using Mouse Interleukin IL-1β and TNF-α ELISA kits (R&D Systems, Minneapolis, MN, USA).

### Immunofluorescence (IF)

After general anesthesia, cardiac perfusion was performed using normal saline and 4% PFA. The lumbar spinal cord was isolated and immersed in 4%PFA for 4-6 h and then dehydrated with 30% sucrose until the tissue sank to the bottom. After freezing, sliced, washed by PBS, and blocked with 5% donkey serum at room temperature for 1 h, the slides were incubated with primary antibodies (GFAP (diluted 1:400; Cell Signaling Technology, USA), Iba1 (diluted 1:100; Cell Signaling Technology, USA)) at 4° C overnight. In the next day, the slides were washed with TBST for 8 min/3 times, and then incubated with goat anti-rabbit recombinant secondary antibody (diluted 1:200; Proteintech, USA) for 1 hour at room temperature in the dark. After washed by TBST for 8 min/3 times, the slides were sealed with DAPI-containing antifade reagents, and then photographed by fluorescence microscope.

### Western blot

Total protein was extracted from tissues or cells by using RIPA lysis buffer (supplemented by phosphatase inhibitors and protease inhibitors). And the protein was quantified by BCA kit and then boiled denaturation. The lysates were separated on 12.5% sodium dodecyl sulphate-polyacrylamide (SDS-PAGE) gels, and then was transferred to 0.22-μm PVDF membranes by electroblotting. The membranes were blocked with 2% BSA at room temperature for 1 h, and then were incubated with relative primary antibodies at 4° C overnight. In the next day, the membranes were washed with TBST for 8 min/3 times, and then were incubated with secondary antibody at room temperature for 1 h. After being washed with TBST for 8 min/3 times, ECL luminescent solution was applied on the membranes for imaging collection.

### Statistical analysis

All data were analyzed and graphed via GraphPad Prism 8.0. IF and WB results were processed by Image J software to measure fluorescence or gray values. Briefly, in the IF analysis, grayscale intensity of IF images was quantified using ImageJ software (National Institutes of Health, Bethesda, MD, USA). Firstly, immunofluorescence images were converted to 8-bit grayscale format. Regions of interest (ROIs) were delineated using the Rectangle or Ellipse selection tools. Mean grayscale intensity values were then measured for each ROI. This process was repeated for all ROIs within the image. Consistent settings and conditions were maintained throughout image capture and analysis to ensure accuracy and reliability of the results. All data were shown as mean ± standard error (± S.E.M.), behavioral data were analyzed by two-way ANOVA [47, 48], other data were analyzed by one-way ANOVA, and two-sample t-test was used for data between two groups. The criterion was that when the test level P < 0.05, the difference was statistically significant.
